# A Novel Metered Dose Transdermal Spray Formulation for Oxybutynin

**DOI:** 10.4103/0250-474X.49094

**Published:** 2008

**Authors:** A. Bakshi, A. Bajaj, G. Malhotra, M. Madan, N. Amrutiya

**Affiliations:** C. U. Shah College of Pharmacy, SNDT Women's University, Santacruz (W), Mumbai-400 049, India

**Keywords:** Transdermal spray, *in vitro* release, kinetics, oxybutynin

## Abstract

The objective of the present work was to develop a metered dose spray formulation for transdermal delivery of oxybutynin and to carry out the in vitro characterization of the optimized formulation. Oxybutynin release from a series of ethanol/acetone/methylal based formulations was assessed *in vitro* and the developed formulation was used for delivery from a metered dose spray. Various qualitative and quantitative parameters like spray pattern, particle size distribution, pH, evaporation time, pump seal efficiency test, average weight per metered dose, content per spray and content uniformity were evaluated. The different film forming agents were assessed and carbopol (0.5%) and lutrol (0.1%) were found to give good clarity of solution, evaporation rate, spray pattern and tackiness of the film. Diffusion studies of the optimized formulations through the semipermeable membrane showed the release of drug to the extent of almost 50% over a period of 24 h. Stability studies were conducted as per ICH guidelines and indicated that formulations were stable. Skin irritation studies were performed using rabbit as an animal model. The results obtained show that the metered dose transdermal spray formulation can be a promising and innovative therapeutic system for the transdermal administration of oxybutynin.

Transdermal delivery is now a well accepted means for systemic delivery of drugs with low oral bioavailability, high first pass effect and gastrointestinal irritation^1^. Efficacious and safe levels of the drugs through percutaneous absorption are obtained systemically from formulations like transdermal patches, gels and sprays^2^. A transdermal patch is placed on the skin to deliver a sustained release dose of medication through the skin^3^. This system provides controlled release of the drug in the patient, and enables a steady blood-level profile, leading to reduced systemic side effects and, sometimes, improved efficacy over other dosage forms^4^–^6^. A less elegant but effective alternative to a transdermal patch system is a conventional topical vehicle such as a gel^7^. Such a formulation shows a clinically equivalent performance to that of a patch with lesser skin irritation and good compliance.

Metered dose transdermal spray (MDTS) is better alternative to both the patch and gel systems, as the sprays form the film that is quick drying, easy to use, well tolerated and non-occlusive^8^. The MDTS formulations are topical aerosols formulated as single phase solutions consisting of drug, polymers and penetration enhancers^9^. New metered dose transdermal sprays, commercialized by Acrux Limited, have enhanced the current growth rate of transdermal drug delivery (TDD) systems by broadening the patient acceptance and compliance.

Overactive bladder is a condition characterized by urinary frequency and urgency, with or without incontinence and nocturia^10^. Several antimuscarinic agents are currently available for the pharmacological treatment of overactive bladder in adults, including oxybutynin (OXY), tolterodine, darifenacin and solifenacin^11^. Oral administration of oxybutynin produces a high incidence of anticholinergic adverse events, such as dry mouth. This is due to the high serum concentration of the active metabolite *N*-desethyloxybutynin that follows hepatic first pass metabolism in the gut and liver^12^. Clinical trials showed that a transdermal delivery of oxybutynin may be associated with a lower incidence of anticholinergic adverse events compared with both the immediate-release and the extended-release oral formulations^13^. The aim of this work was to realize and test the MDTS formulations for oxybutynin release *in vitro* using rabbit ear skin as the semipermeable membrane. The developed spray formulations would be evaluated for the performance characteristics like spray pattern, particle size distribution, evaporation time, average weight per metered dose and content per spray. It was also proposed to carry out safety studies using rabbit as an animal model.

## MATERIALS AND METHODS

Oxybutynin was obtained as a gift sample from Cipla Ltd, Mumbai. Carbopol and Lutrol were purchased from Noveon and BASF, Mumbai, respectively. Glyceryl monooleate and myristyl lactate, were purchased from Brink Chemicals Private Limited, Mumbai. Cyclomethicone was procured from International Drug and Chemical Company, Mumbai. Approval of the Institutional Animal Ethical Committee was obtained prior to the commencement of skin irritation studies.

### Selection of excipients:

A series of batches were prepared using different aqueous and non aqueous solvents, co-solvents, permeation enhancers and polymers in varying concentrations. Ethanol, acetone, methylal and dimethoxymethane were selected as solvents for formulating MDTS due to the good solubility of polymers in these solvents. Preformulation studies showed that solvent system consisting of ethanol: acetone: methylal in the ratio 2:1:2 exhibited desired spray patterns and high dispersibility of the polymers. Cyclomethicone is a volatile, low viscosity silicon fluid and was used as a cosolvent. Permeation enhancers like monoglycerides and lactate esters were incorporated for transdermal delivery of oxybutynin. Polymers with known solubility in ethanol and acetone and having desired film forming, gelling and coating properties were selected for the study. Various carbomers and poloxamers were used. Table 1 reports the compositions of the mixtures used for MDTS formulations in the finished product. The formulations prepared were evaluated for their oxybutynin content, expressed as %w/w.

**TABLE 1:** COMPOSITIONS OF THE PREPARED BATCHES OF OXYBUTYNIN MDTS FORMULATIONS.

Ingredients (%w/w)	F1	F2	F3	F4	F5	F6	F7	F8	F9	F10	F11
Oxybutynin	10	10	10	10	10	10	10	10	10	10	10
Glyceryl mono oleate	-	0.5	0.5	0.5	0.5	0.5	0.5	0.5	0.5	-	-
Myristyl lactate	-	0.5	0.5	0.5	0.5	0.5	0.5	0.5	0.5	-	-
Water	-	0.5	0.5	0.5	0.5	0.5	0.5	0.5	0.5	0.5	0.5
Cyclomethicone	-	-	-	-	-	-	-	0.5	0.5	-	-
Carbopol-940	-	-	0.50	-	1.0	0.25	-	-	0.50	-	0.50
Lutrol F-127	-	0.10	-	0.20	-	-	0.05	0.10	-	0.10	-
Ethanol:acetone:methylal(2:1:2)	q.s.	q.s.	q.s.	q.s.	q.s.	q.s.	q.s.	q.s.	q.s.	q.s.	q.s.

*Formulation F1 did not contain any film forming agent and was used as a reference. Formulation F2 and F3 were found to be optimized formulations. The effect of increased and decreased concentrations of film forming agents was revealed by formulations F4, F5, F6 and F7. The presence of cyclomethicone as a cosolvent did not affect the film formation from optimized MDTS formulations. Absence of penetration enhancers led to very less drug release as shown by formulations F10 and F11.

### Selection of containers, spray pumps and actuators:

Specially designed glass bottles with a ‘V’ shape carved at the base were utilized for filling the transdermal spray formulations. The spray pumps were selected based on the required dose of the drug and physico-chemical compatibility of components with formulations. Two types of pumps, VP3 and VP7 (Valois Ltd., Mumbai) were tried for propellant free delivery systems. VP7 type pump was selected on the basis of required dose to be actuated. Six different types of topical spray actuators viz. 25140BGP (100 mm), 25140BGP (400 mm), 165AGPC (8PH11038), 155 A GPC (8PH11633), CB 18131 NA+B25 and 102 B (marketed by Valois Ltd., Mumbai) were tried. The actuator type 165AGPC was selected as it is specially designed for 100 μl pumps. Spray properties of different actuators were studied using ethanol:acetone:methylal (2:1:2) solvent system that contained Lutrol F-127 as film former. The actuator with the actuating volume of 100 μl was selected and used for further investigation.

### Formulation development of topical spray preparation:

The MDTS formulations were developed as topical solutions made up of volatile and non volatile vehicles containing the drugs and polymers dissolved in a single phase. The polymeric spray systems were prepared by incorporating the polymers and permeation enhancers into the solvent system. The drug was accurately weighed in specially designed glass bottles and the total weight of the system was adjusted with the polymeric solvents in such a way that desired amount of drug could be obtained after each actuation.

### Characterization of developed MDTS formulations:

The quality control testing of the MDTS formulations was carried out mainly to optimize the transdermal spray delivery system and to improve the efficiency of the delivery of the active ingredients. The qualitative tests performed for the MDTS formulations included the evaluation of spray pattern, particle size distribution, evaporation time, effectiveness of pump seal and pH. The spray pattern was assessed by delivering the spray through the MDTS onto a glass plate containing activated silica gel-dye mixture. The formulation was held at a distance of 2.5-3.0 cm from the plate. The spots formed as a result of spray testing were observed under UV light and their diameters were measured. This parameter was also checked using ethanol sensitive paper. The paper was clipped on a board and the formulation was sprayed on it and the spray pattern was evaluated in the same manner as mentioned for the silica plate.

Particle size distribution was determined by optical microscopy. The formulation was sprayed on a glass slide and at least 100 particles in 25 different fields were measured under a binocular Labomed vision 2000 microscope of magnification 40X. Evaporation time is the time required for the spray film to dry and it was estimated by spraying the formulation on ethanol sensitive paper and noting down the drying time. Effectiveness of the pump seal and its ability to store the contents of the product was evaluated by Leak test/Pump seal efficiency test. The filled containers under test were placed in the upright position at 30° for specified period of time. The containers were weighed before and after the test period. The change in the weight of the container was noted down and the leakage rates were calculated. pH of the developed formulations was determined using a pH meter (DBK Instruments, Mumbai). The clarity and tackiness of the sprayed films were also noted.

The following quantitative tests for the MDTS formulations were also performed. Average weight per metered dose is an important quantitative parameter to be evaluated. The initial weight of the container was recorded. Five successive deliveries were sprayed from the MDTS and the containers were weighed again. The difference in the initial and final weight of the containers divided by the number of deliveries sprayed from the containers was used to determine the average weight per metered dose. The capacity of the pumps utilized for MDTS was 100 μl.

The content per spray was determined by firing ten sprays in a beaker containing acetonitrile. This solution was shaken for 10 m and the drug content was analyzed by HPLC^14^ (Tosoh, Japan). Briefly, a 35:65 (v/v) mixture of acetonitrile and phosphate buffer (0.02 M KH_2_PO_4_, 0.005 M sodium hexane sulfonate, 0.3% v/v triethylamine, adjusted to pH 4.5 with phosphoric acid) was used as mobile phase at a flow rate of 1.2 ml/min. A sample volume of 20 μL was injected onto a Cosmosil 5CN-MS column (4.6×250 mm, particle size 5 μm), maintained at 40° in a column oven, with a CN guard column. The analysis was performed at the wavelength of 220 nm. Content uniformity was assessed by analyzing the drug content in 1^st^, 10^th^, 15^th^, 30^th^ and 40^th^ doses emitted from the pump. The results are shown in Table 2.

**TABLE 2:** CHARACTERIZATION OF REFERENCE FORMULATION AND OPTIMIZED OXYBUTYNIN MDTS FORMULATIONS

Parameter	Results		
	
	$F1	$$F2	$$$F3
*Spray pattern	Less uniform films.	Better uniformity of films and more spherical spots.	Better uniformity of films and more spherical spots.
*Mean particle size (μ) (±SD)	30 (±1.4)	38 (±0.75)	45 (±2.6)
*Evaporation time	80-85 s	65-70 s	65-70 s
*Leakage from container	No leakage	No leakage	No leakage
*pH	5.3-5.5	6.0-6.5	5.0-5.5
**Average weight of the formulation per metered dose (mg±SD)	90 (±1.645)	90 (±0.68)	90 (±0.97)
**Drug content per spray (%) (±SD)	104.06 (±1.721)	105.01 (±0.13)	102.78 (±1.153)
**Content uniformity (%) (±SD)	101.65 (±1.569)	103.78 (±0.462)	104.02 (±1.432)

* Qualitative tests carried out for optimized MDTS formulations (n=3)

** Quantitative tests carried out for MDTS formulations (n=6)

$ Reference formulation

$$ Formulation containing Lutrol F-127 as the film former

$$$ Formulation containing Carbopol as the film former.

### *In vitro* skin permeation experiments:

The skin peeled from rabbit ear was used for *in vitro* permeation studies. The dorsal skin without adhering subcutaneous fat was mounted on a modified Franz diffusion cell with a surface area of 7.0 cm^2^. The receptor phase was PBS at pH 7.4 thermostated at 37° and stirred with a Teflon coated magnetic stirrer. At predetermined time intervals, the receptor solution was sampled and analyzed by HPLC for determination of drug permeated. Each permeation experiment was repeated at least 6 times. Percentage cumulative amounts of drug penetrated were plotted as a function of time. The results were expressed as the mean±SEM. Statistical significance of the results was ascertained using Student's t-test at a probability level of p≤ 0.05.

### Stability studies:

The developed formulations were stored for stability testing as per ICH guidelines. The chemical stability of the formulations was assessed by estimation of the percent drug remaining in the formulations, drug release pattern and physical stability was evaluated by monitoring any change in pH, appearance, spray pattern, leakage rate and average weight per actuation.

### Skin irritation studies:

Draize patch test was carried out using white rabbits (New Zealand strain) as the animal model. The optimized formulation was sprayed on the patch of preshaved skin and occluded with adhesive tapes and resulting reactions such as erythema and edema were scored after 24 h.

## RESULTS AND DISCUSSION

The optimized formulations were selected based on the clarity of the solution, evaporation rate, spray pattern and tackiness of the film formed. Formulation F1 was a simple drug solution in solvent system and was used as a reference. The formulations F2 and F3 contained Lutrol F-127 and Carbopol-940 as polymers, respectively^15^. Glyceryl monooleate and myristyl lactate were incorporated as permeation enhancers in F2 and F3 formulations^16^. The films formed by the transdermal sprays, composed of film forming agent and penetration enhancers, were transparent and cohesive. The organic solvents in the formulations evaporated quickly leaving behind a thin film that adhered to the skin for periods up to 24 h. Comparing the physicochemical characteristics of the films, spray patterns and drug content, formulations F2 and F3 were found to be better as compared to other formulations and were subjected for further characterization. The detailed results of qualitative and quantitative tests for reference formulation and the optimized formulations (F2 and F3) are summarized in Table 2.

When the spray pattern was evaluated using the TLC plate method, the spots obtained from all the formulations were violet pink in color, when observed under UV light. Spray on the ethanol sensitive paper exhibited blue coloration against a red background indicating the nature of spray pattern. The spray pattern was found to be affected by the size and shape of actuator orifice as well as by the capacity of pump used. Diameters of the spots were found to be increased in the range of 3.08-3.45 cm, as the microlitre capacity of the pumps was increased in the range 50-100 μl. Formulations F2 and F3 showed good spray patterns in terms of uniform and spherical spots due to the flexible and cohesive film forming nature of the polymers used.

Majority of the formulations delivered the droplets within desired size range of 5-50 μm for film formation on the skin. Formulation F1 being a simple drug solution required 80-85 s to dry and formed a film as compared to the optimized MDTS formulations (65-70 s). The reason may be attributed to the absence of film forming agent in formulation F1. Dry films formed by the formulations F2 and F3 were more acceptable as compared to those obtained from F1. This test indicated the effectiveness of the solvents in drying the film. No leakage was observed from the MDTS containers when placed in the upright position at 30° for 3 d. Formulations containing Lutrol F-127 as a polymer showed pH in the range of 6.0-6.5, whereas pH of 5.0-5.5 was observed with the formulations containing Carbopol 940 as polymer thus mimicking the skin pH. The 100 μl pumps delivered average weight of 90±2.0 mg per actuation (n=5). There was no statistically significant variation in the amount emitted per actuation indicating the effectiveness of the pump system in delivering reproducible amounts of the formulation per actuation.

The dose of the drug delivered per actuation of the pump was within the range 101-106%. Formulations F1, F2 and F3 showed average drug contents per spray of 104.06±1.721%, 105.01±0.13% and 102.78±1.153%, respectively. This test indicates the amount of the therapeutically active ingredient delivered per metered spray from the metered dose containers. Formulation F2 was found to show better uniformity in terms of content per spray. Content uniformity was assessed for I^st^, 10^th^, 15^th^, 30^th^ and 40^th^ doses and the results indicated that the uniform drug content per actuation was maintained up to 40^th^ dose for oxybutynin. We inferred that the formulation F2 had more uniformity of the dosage of the active ingredient delivered through the metered pump compared to formulations F1 and F3 due to the presence of Lutrol F-127.

**Fig. 1:**
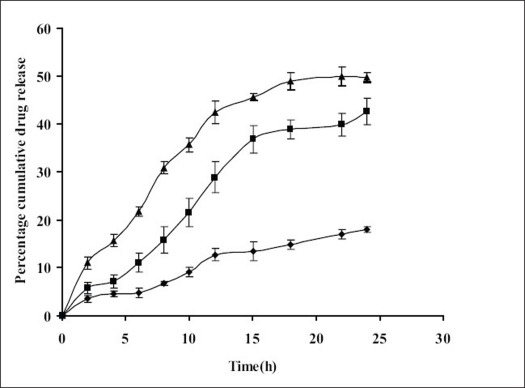
Effect of film formers on *in vitro* release profile of oxybutynin Permeation of drug from reference solution (F1, –◆–) and from MDTS formulations containing Carbopol (F3, –■–) and Lutrol F-127 (F2, –▲–) as the film formers, respectively. Each value is mean±SEM of (n=3). The formulations F2 and F3 showed significantly higher release (*P*≤0.05) of drug as compared to formulation F1

**Fig. 2:**
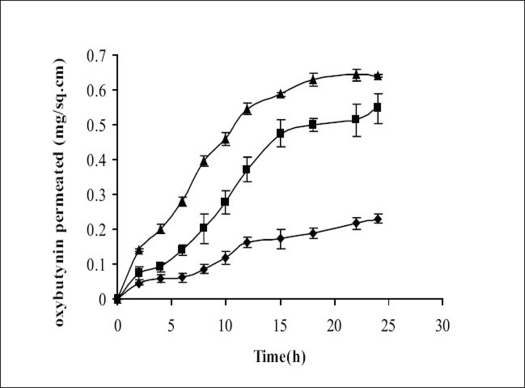
Permeation of oxybutynin from reference solution and from MDTS formulations Rabbit ear skin was used as the diffusion membrane. Release profile from reference solution (–◆–) and from MDTS Formulations containing Carbopol (F3, –■–) and Lutrol F-127 (F2, –▲–). Each value is mean±SEM of (n=3).

Permeation properties of the drug were characterized initially by conducting diffusion studies on the simple organic drug solution (F1). The diffusion studies from the optimized formulations F2 and F3 were also carried out over a period of 24 h. Rabbit ear skin was used as animal model skin, because of its similarity with human skin *in vitro*. As indicated from figs. 1 and 2, the release of oxybutynin from the optimized formulations was found to be 45-50% in 24h.

Different kinetic models (First order release, Higuchi equation and zero order release) were employed to fit the data relating to the kinetics of the release of oxybutynin from MDTS formulations. The results (Table 3) showed that on the basis of the highest R^2^ values, the release kinetics fits the zero order kinetic model for formulation F3 and root time kinetics for formulation F2. Analysis of variance showed that these correlations are statistically significant (*P*≤0.05). As shown in fig. 2, a linear relationship was obtained between Q (cumulative amount of drug penetrated through the unit surface area of the skin) and time up to 24 h. The formulations F2 and F3 showed significantly higher release (*P*≤0.05) of drug as compared to formulation F1, because of the presence of penetration enhancers and film forming agents. Formulations F2 and F3 contained glyceryl mono oleate and myristyl lactate as the permeation enhancers leading to more permeation of drug into the skin. Lutrol F-127 and Carbopol, used as the film forming agents in formulations F2 and F3, respectively, helped in the formation of thin film in direct contact of the skin thus releasing the drug for a longer period of time. Stability studies showed the formulations to be stable in terms of physicochemical parameters, drug content and drug release, under all the storage conditions (Table 4).

**TABLE 3:** PERMEATION PARAMETERS OF OXYBUTYNIN ACROSS RABBIT EAR SKIN

Formulation	Flux (mg/cm^2^.h)	Intercept	R^2^	*Q_24_ (mg/cm^2^)
F1	0.0095	0.0178	0.9655	0.2312
F2	0.0264	0.1205	0.9608	0.6405
F3	0.0244	0.0223	0.9494	0.5469

* Amount of drug released per unit surface area after 24 h. Formulations F1 and F3 showed zero order kinetic model while formulation F2 showed root time kinetics with R^2^ value of 0.9608. [Formulations F2 and F3 (containing film formers and penetration enhancers) showed more flux and Q_24_ value for the drug as compared to Formulation F1].

**TABLE 4:** STABILITY STUDIES DATA OF THE OPTIMIZED MDTS FORMULATIONS AT DIFFERENT TEMPERATURE CONDITIONS, AFTER THREE MONTHS (N=3)

Parameters evaluated	Stability temperature		

	8±2°	25±2°	37±2°
Formulation F2			
Appearance	Clear yellowish	Clear yellowish	Clear yellowish
pH (±SD)	6.2±0.05	6.3±0.001	6.2±0.02
Spray pattern	Uniform and spherical	Uniform and spherical	Uniform and spherical
Leakage rate	0.01%	0.01%	0.02%
Average weight per actuation (mg±SD)	90.0±0.56	90.76±1.24	91.42±1.54
Percent drug remaining (±SD)	100.07±0.45	99.75±1.31	99.52±1.07
Cumulative drug release (%±SEM)	53.63±0.72	52.20±0.82	54.62±0.34
Formulation F3			
Appearance	Clear yellowish	Clear yellowish	Clear yellowish
pH (±SD)	5.0±0.008	5.3±0.02	5.0±0.05
Spray pattern	Uniform and spherical	Uniform and spherical	Uniform and spherical
Leakage rate	0.01%	0.01%	0.02%
Average weight per actuation (mg±SD)	90.0±1.12	91.11±0.93	91.74±1.29
Percent drug remaining (±SD)	101.14±1.03	100.59±0.99	99.82±1.51
Cumulative drug release (%±SEM)	45.56±0.53	43.94±1.39	47.48±0.87

No erythema or edema were found to occur in the primary skin irritation studies of the optimized formulations on the rabbits hence found to be safe and non irritant for transdermal application. The formulation developed was more efficient for a period of 24 h after application. However results need to be confirmed by pharmacokinetic studies.

From the results obtained in the present work, it can be concluded that the MDTS formulations for oxybutynin can be an innovative and promising approach for the transdermal administration of oxybutynin. The diffusion studies indicated that the permeation of MDTS formulations through the skin was much higher as compared to the diffusion of simple organic solution of the drug. Such a controlled release MDTS formulation will have better patient compliance and acceptability.
